# Organ-On-A-Chip *in vitro* Models of the Brain and the Blood-Brain Barrier and Their Value to Study the Microbiota-Gut-Brain Axis in Neurodegeneration

**DOI:** 10.3389/fbioe.2019.00435

**Published:** 2020-01-10

**Authors:** Ilaria Raimondi, Luca Izzo, Marta Tunesi, Manola Comar, Diego Albani, Carmen Giordano

**Affiliations:** ^1^Department of Chemistry, Materials and Chemical Engineering “Giulio Natta”, Politecnico di Milano, Milan, Italy; ^2^SSD of Advanced Translational Microbiology, IRCCS “Burlo Garofolo”, Department of Medical Sciences (DMS), University of Trieste, Trieste, Italy; ^3^Department of Neuroscience, Istituto di Ricerche Farmacologiche Mario Negri IRCCS, Milan, Italy

**Keywords:** microbiota-gut-brain axis, neurodegenerative diseases, *in vitro* modeling, microfluidics, brain, blood-brain barrier

## Abstract

We are accumulating evidence that intestinal microflora, collectively named gut microbiota, can alter brain pathophysiology, but researchers have just begun to discover the mechanisms of this bidirectional connection (often referred to as microbiota-gut-brain axis, MGBA). The most noticeable hypothesis for a pathological action of gut microbiota on the brain is based on microbial release of soluble neurotransmitters, hormones, immune molecules and neuroactive metabolites, but this complex scenario requires reliable and controllable tools for its causal demonstration. Thanks to three-dimensional (3D) cultures and microfluidics, engineered in *vitro* models could improve the scientific knowledge in this field, also from a therapeutic perspective. This review briefly retraces the main discoveries linking the activity of gut microbiota to prevalent brain neurodegenerative disorders, and then provides a deep insight into the state-of-the-art for *in vitro* modeling of the brain and the blood-brain barrier (BBB), two key players of the MGBA. Several brain and BBB microfluidic devices have already been developed to implement organ-on-a-chip solutions, but some limitations still exist. Future developments of organ-on-a-chip tools to model the MGBA will require an interdisciplinary approach and the synergy with cutting-edge technologies (for instance, bioprinting) to achieve multi-organ platforms and support basic research, also for the development of new therapies against neurodegenerative diseases.

## The Microbiota-Gut-Brain Axis and Its Involvement in Neurological Diseases

Research on microbiota is rooted in Antonie van Leeuwenhoek's pioneering work (XVII century) (Bardell, 1983), but in recent years it has aroused a growing interest. In particular, the gut microbiota has strongly emerged as a key player both in physiological and pathological conditions (Jia et al., 2008). The gut microbiota is a complex and dynamic population of tens of trillions of microbes residing in the gastrointestinal (GI) tract, with a mutualistic relationship with the host.

In homeostatic conditions, it interacts with the intestinal mucosa and maintains its integrity (Groschwitz and Hogan, 2009), contributes to the development and maturation of the endocrine system (Farzi et al., 2018), influences the migration, functions and population of the immune system and produces neuroendocrine hormones and neuroactive compounds (Holzer and Farzi, 2014). It also influences microglial activation and development (Erny et al., 2015; Fung et al., 2017), astrocyte functions (Fung et al., 2017), the integrity of the blood-brain barrier (BBB) (Braniste et al., 2014; Michel and Prat, 2016), the production of neurotransmitters (Luczynski et al., 2016), and neuroimmune activation (Sampson et al., 2016; Dinan and Cryan, 2017).

The determination and formation of the gut microbiota involves several factors, like stress, diet, smoking, surgery and environment (Biedermann et al., 2013; Tyakht et al., 2013; Jiang et al., 2015; Rodríguez et al., 2015). Its composition may change during life (Thursby and Juge, 2017) and its maintenance during development and maturation is important to prevent inflammation and disorders. For instance, alterations in resident gut microbiota (*dysbiosis*) activate the immune system (including T-cells) and increase the levels of inflammatory mediators, the permeability of the gut barrier (Groschwitz and Hogan, 2009; Rea et al., 2016) and mucus production, which in turn promote neuroinflammation, neural injury and neurodegeneration (Kowalski and Mulak, 2019).

Möhle et al. have studied the influence of gut dysbiosis on hippocampal neurogenesis. During behavioral tests, they have observed alterations in germ-free (GF) and specific pathogen-free (SPF) mice treated with antibiotics (Al-Asmakh and Zadjali, 2015; Möhle et al., 2016). GF mice are free of all microorganisms and they represent a powerful tool to investigate how microbes affect the host and the relationship between microbiome and disease (Wos-Oxley et al., 2012). Oppositely, SPF mice are free of a defined list of mouse pathogens, depending on the husbandry methods (Hirayama et al., 1990). With respect to controls with gut microbiota, other groups have found alterations in BBB functions and cortical myelination (Hoban et al., 2013; Braniste et al., 2014), defects in gut motility, progressive deficits in fine and gross motor skills, reduced microglial activation, the presence of α-syn inclusions and motor deficits (Sampson et al., 2016).

There is increasing evidence that dysbiosis is involved in several pathological states, including epilepsy (Iannone et al., 2019), inflammatory bowel disease (Tung et al., 2011; Moser et al., 2018), anxiety and depression (Jia et al., 2008; Wang and Kasper, 2014; Foster et al., 2017), autism spectrum disorders (Mayer et al., 2014; Li and Zhou, 2016; Yarandi et al., 2016), schizophrenia (Nemani et al., 2015), and neurodegenerative disorders (Sarkar and Banerjee, 2019), such as Alzheimer's disease (AD) (Vogt et al., 2017; Van Giau et al., 2018), Parkinson's disease (PD) (Foster and McVey Neufeld, 2013; Nemani et al., 2015; Sampson et al., 2016; Harach et al., 2017) and multiple sclerosis (MS) (Cekanaviciute et al., 2017; Mowry and Glenn, 2018). In fact, the bidirectional microbiota-gut-brain communication (the so-called *microbiota-gut-brain axis*, MGBA) is not limited to digestive functions and satiety, but the gut microbiota also influences behavior and cognitive abilities (Di Meo et al., 2018; Cryan et al., 2019).

AD leads to a progressive and irreversible decline in memory and a deterioration of cognitive abilities. It involves the destruction of nerve cells and neural connections in the cerebral cortex, with a significant loss of brain mass. Its hallmarks are intracellular neurofibrillary tangles (Arriagada et al., 1992; Reitz et al., 2011) and extracellular senile plaques mainly composed of β-amyloid (Aβ) (Scheuner et al., 1996; Shankar et al., 2008). Kowalski and Mulak reviewed the results in both animal models and clinical trials, supporting the evidence of a correlation between dysbiosis and AD (Kowalski and Mulak, 2019). For instance, APP/PS1 mice are a mouse model of early-onset AD. They are double-transgenic mice expressing the KM670/671NL Swedish mutation of human amyloid precursor protein and the L166P mutation of human presenilin 1 under the control of thymocyte differentiation antigen 1 (Thy-1) promoter and they show an age-dependent accumulation of parenchymal Aβ plaques, with minimal vascular Aβ. With respect to age-matched wild-type controls, *Firmicutes* and *Bacteroidetes* increased in APP/PS1 mice, while *Allobaculum* and *Akkermansia* decreased (Harach et al., 2017).

PD affects the dopaminergic neurons in the *substantia nigra* and impairs motor control. Its pathological hallmark is the deposition of α-synuclein (α-syn) in susceptible neurons in the form of Lewy bodies and Lewy neurites (Dauer and Przedborski, 2003). This accumulation also leads to an increase in intestinal permeability and to the possible translocation of bacterial or microbial inflammatory compounds into the bloodstream (Forsyth et al., 2011; Perez-Pardo et al., 2017). Some studies have hypothesized that α-syn is involved in GI dysfunctions and damage to enteric neurons by showing its accumulation in the enteric nervous system (Braak et al., 2006; Forsyth et al., 2011; Gold et al., 2013; Gelpi et al., 2014), whose neurons are specifically associated with the GI tract to control several activities, such as mucosal transport, secretion and modulation of immune and endocrine functions (Gold et al., 2013; Sánchez-Ferro et al., 2015).

MS is an inflammatory, autoimmune disease characterized by damage to the myelin sheaths, axonal degeneration, atrophy of nerve fibers and progressive neuronal loss. Recent studies have highlighted the possibility that some bacterial taxa are significantly associated with MS. For instance, *Akkermansia muciniphila* and *Acinetobacter calcoaceticus* (known to induce proinflammatory responses in human peripheral blood mononuclear cells and mono-colonized mice) were increased in MS patients, while *Parabacteroides distasonis* (which stimulates anti-inflammatory interleukins in humans and mouse models) was reduced (Cekanaviciute et al., 2017; Mowry and Glenn, 2018). In addition, they have suggested an effect of probiotics, with an increase of taxa like *Lactobacillus, Streptococcus*, and *Bifidobacterium* in both controls and MS patients (Tankou et al., 2018).

The neurodegenerative disorders reported above are just examples of how the microbiota can impact on the brain and its severe and long-lasting diseases and researchers are now beginning to investigate how this may happen in terms of pathophysiological mechanisms in a wide list of brain diseases (De Caro et al., 2019a,b).

## Communication Pathways in the MGBA

The bidirectional communication among the microbiota, the gut and the brain exploits neural messages from the vagus nerve and spinal afferent neurons, the release of microbial factors, gut hormones and cytokines from the immune system (Holzer and Farzi, 2014; El Aidy et al., 2015; Mayer et al., 2015; Sherwin et al., 2016; Lerner et al., 2017; Farzi et al., 2018). It takes place by distinct pathways involving the autonomic nervous system (ANS), the enteric nervous system (ENS), the hypothalamic-pituitary-adrenal axis (HPA), the neuroimmune system and metabolites translocating from the intestinal mucosa into the bloodstream (Grenham et al., 2011; Bhattacharjee and Lukiw, 2013; Borre et al., 2014; Daulatzai, 2014; Stilling et al., 2014).

The ANS is a component of the peripheral nervous system and divides into sympathetic and parasympathetic system. It connects visceral response and central activity and it regulates mucus secretion and motility, gastric secretions, the production of bicarbonate and gut antimicrobial peptides, the absorption and release of fluids by epithelial cells, the permeability of intestinal cells, and mucosal immune response against pathogens (Tougas, 1999; Mayer, 2011; Martin et al., 2018). It transmits efferent signals from the central nervous system (CNS) to the intestinal walls and afferent signals from the intestinal lumen to the CNS through enteric, spinal and vagal pathways (Carabotti et al., 2015). The vagal afferent fibers spread to all the layers of the intestinal wall, but they do not cross the innermost layer (Wang and Powley, 2007). They receive signals from the microbiota only indirectly, by released metabolites or bacterial compounds and the epithelial cells transducing luminal signals (Bonaz et al., 2018). The communication pathway based on the ANS can influence the expression of γ-aminobutyric acid (GABA) receptors and reduce anxiety and depressive behavior (Bravo et al., 2011). Thanks to the autonomic-related projection of the neurons, stress inhibits the vagus nerve and stimulates the sympathetic system (Taché and Bonaz, 2007; Wood and Woods, 2007). After acute stress, there is an increase in the release of pro-inflammatory cytokines (Marsland et al., 2017), while the afferent fibers of the vagus nerve have anti-inflammatory properties due to the stimulation and involvement of several pathways. The influence of these opposite effects can cause the loss of the protective effect, leading to dysbiosis and disruption of the homeostasis of the epithelial barrier (Bonaz et al., 2013). So far, *in vitro* models able to reproduce this anatomical pathway are not available, mainly because it is strictly connected with other communication pathways, the connections are complex and it involves the release of different factors from the gut.

The ENS is one of the main divisions of the ANS, but from a functional point of view, it can be regarded as a separate part. It regulates the motor and secretory functions of the GI, and it is involved in the maintenance of the GI homeostasis by allowing the crosstalk among the brain, the gut microbiota, the endocrine and the immune systems. It is the target of bacterial metabolites, so individual microbes and strains could influence its activity and neurochemistry. Ion and fluid secretion from the gut, epithelial barrier and mucus layer protect the ENS from lumen content and separate from the gut microbiota (Saulnier et al., 2013). In GF mice on postnatal day 3, the neurochemical profile and function of the ENS decrease during the development of the enteric neural network compared to SPF mice or dams colonized with microbiota (Collins et al., 2014; Lomasney et al., 2014), suggesting that microbiota influences the development of the ENS (Hyland and Cryan, 2016). The ENS is also referred to as a second brain, because it is involved in the production of neurotransmitters. Enteroendocrine cells of the GI tract produce about 90–95% of the total body 5-hydroxytryptamine (5-HT, serotonin) (Kim and Camilleri, 2000), that plays an important role in the regulation of GI motility and secretion. Recent studies in GF mice colonized with physiological mouse microbiota have reported that the production of neuronal and mucosal 5-HT and the proliferation of enteric neuronal progenitors correlate to a modification of the neuroanatomy of the ENS and an increase in intestinal trafficking (Grider and Piland, 2007; De Vadder et al., 2018). An *in vitro* system modeling the ENS is available. It exploits a Transwell®-based culture plate and the co-culture of murine small intestinal stem cells, ENS neurons and glia or subepithelial myofibroblasts and it is able to catch the important relationship between enteric population and the functions of the intestinal barrier (Schlieve et al., 2017; Workman et al., 2017; Puzan et al., 2018).

The HPA is the main neuroendocrine system. It regulates different body processes, such as digestion, energy, immune system, and emotions (Sudo, 2012) and it coordinates the adaptive responses to stress by the release of hormones (Tsigos and Chrousos, 2002). During stress, the composition of the gut microbiota changes in response to the release of neuroendocrine hormones (e.g., norepinephrine, dopamine). They increase the growth of Gram-negative bacteria (Lyte et al., 2011), with a consequent increase in the permeability of the intestinal barrier, inducing an inflammatory response and bacterial translocation across the intestinal lumen (De Punder and Pruimboom, 2015). During stress, GF mice release more corticosterone and adrenocorticotrophic hormone than SPF ones, indicating a higher degree of anxiety and stress (Foster and McVey Neufeld, 2013). The gut microbiota also influences the limbic system by producing serotonin and related metabolites (Clarke et al., 2013). Several *in vitro* studies have focused on modeling the HPA and its role. Generally, they are computational (Hosseinichimeh et al., 2015), while experiments have involved treatments with hormones and the evaluation of their effect on cell populations (Al-Asmakh and Zadjali, 2015).

The neuroimmune system regulates the interactions between the nervous and immune systems in both physiological and pathological conditions and it protects the brain against pathogens. The endocrine system permits the passage of information from the nervous system to the immune one, while in the opposite direction the communication exploits inflammatory molecules. The pathway based on the neuroimmune system is important in psychiatry and immunology, as alterations may have pathological consequences and trigger several disorders (Dantzer, 2018). *In vivo* studies in neurodegeneration models have proved the influence of the gut microbiota on the development and functions of the immune cells of the CNS. For instance, in GF mice, microglia may react sooner to pathogen exposure, but integration with a complex microbial community lowers this reaction to standard levels (Erny et al., 2015). In AD patients, an increase in the brain levels of inflammation-related proteins and alterations of the circulating levels of anti-inflammatory and pro-inflammatory cytokines can directly affect brain functions, mood, and behavior and elicit neuroinflammation (Rothhammer et al., 2018). Furthermore, alterations of the intestinal microbiota can induce inflammation and aggregation of cerebral Aβ (Pistollato et al., 2016).

The gut microbiota produces different metabolites that translocate, by direct or indirect passage, from the intestinal mucosa to the systemic circulation and may interfere with the BBB homeostasis and potentially contribute to trigger inflammation and neurodegeneration (Logsdon et al., 2018).

In the case of direct passage, specific immune responses mediated by nerve cells and cellular barriers (e.g., the BBB) protect the brain against microbial invasion. These barriers also allow the delivery of nutrients, the removal of metabolites and the protection of the brain from abrupt changes in blood biochemistry (Dando et al., 2014). Their alteration may modify the CNS homeostasis and change the release and expression of cytokines, chemokines and cell adhesion molecules, or induce cytotoxicity and apoptosis (Kim, 2008). In this case, the pathogenic microorganisms may target different regions, depending on the pathway of invasion, the distribution of cellular receptors and the metabolic environment required for their replication (Kristensson, 2011).

In the case of indirect passage, soluble biochemical factors released by microbes (*secretome*) can reach the BBB and then the brain (Matsumoto et al., 2013), showing a neurotoxic effect and altering brain homeostasis (Table 1). *Cyanobacteria* produce β-methylamino-L-alanine (BMAA), a non-protein neurotoxic amino acid that can trigger pathological processes (e.g., protein misfolding and aggregation, oxidative stress) frequently observed in neurodegeneration (Brenner, 2013; Karlsson et al., 2014). Lipopolysaccharide (LPS) has also been found in several neurodegenerative conditions. It is a component of the external cell membrane of Gram-negative bacteria. It induces an immune response and increases the permeability of the BBB (Qin et al., 2007; Tufekci et al., 2011; Asti and Gioglio, 2014). Oppositely, short-chain fatty acids (SCFA, especially butyrate, propionate and acetate) derive from the fermentation of dietary fibers and are involved in GI functions, neuroimmune regulation, and host metabolism. For instance, butyrate promotes the absorption of minerals (Rivière et al., 2016), stimulates mucin production (Finnie et al., 1995; Van den Abbeele et al., 2013), induces the expression of antimicrobial peptides (Ochoa-Zarzosa et al., 2009; Guaní-Guerra et al., 2010), contributes to the regulation of intestinal cell growth and differentiation (Barnard and Warwick, 1993; Schröder et al., 1999), regulates the gut immune system (Furusawa et al., 2013) and increases the expression of tight junction (TJ) proteins in the BBB.

**Table 1:** Bacteria release neuromodulators.

**Neuro-modulators**	**Bacterial genera**	**Brain disease**	**References**
5-Hydroxytryptamine (5-TH)	*Candida, Enterococcus, Escherichia, Streptococcus*	anxiety, depression, MS, PD	Baganz and Blakely, 2013; Mawe and Hoffman, 2013; O'Mahony et al., 2015; Yano et al., 2015
β-Methylamino-L-alanine (BMAA)	*Cyanobacteria*	AD, ALS, PD	Meneely et al., 2016; Delcourt et al., 2017
γ-Aminobutyric (GABA)	*Bifidobacterium, Lactobacillus*	AD, anxiety, depression	Barrett et al., 2012; Lin, 2013; Hu et al., 2016
Acetylcholine	*Lactobacillus*	AD	Wessler and Kirkpatrick, 2008; Wall et al., 2014
Catecholamine (Adrenaline, Dopamine, Noradrenaline)	*Bacillus, Escherichia, Saccharomyces*	ASD, depression, PD, schizophrenia	Mayer and Hsiao, 2017; Mittal et al., 2017; Sugama et al., 2017
Histamine	*Lactobacillus, Lactococcus, Pediococcus, Streptococcus, Enterococcus spp*.	AD, MS	Landete et al., 2008; Thomas et al., 2012; Naddafi and Mirshafiey, 2013; Westfall et al., 2017
Lipopolysaccharide (LPS)	*Gram-negative bacteria*	AD, anxiety, ASD depression, HD, MS, PD, schizophrenia, etc.	Zhao et al., 2017
Short-chain fatty acids (SCFA, e.g., acetate, propionate and butyrate)	*Bacteroides, Bifidobacterium*, *Clostridium, Eubacterium, Lactobacillus, Propionibacterium, Roseburia Prevotella*	AD, ASD, HD, PD	Liu et al., 2015; Verbeke et al., 2015; Bourassa et al., 2016; Ho et al., 2018; Van de Wouw et al., 2018
Gingipains	*Porphyromonas gingivalis*	AD, PD	Adams et al., 2019; Dominy et al., 2019

*Summary of the most important neuropeptides involved in brain disorders such as anxiety, autism, AD, amyotrophic lateral sclerosis, depression, MS, schizophrenia, and PD*.

Further studies in a gut-unrelated microbiota context have highlighted the relevance of indirect passage for neurodegeneration. For instance, research focused on animal models of periodontitis and brain tissues from AD patients have indicated that *Porphyromonas gingivalis* (a Gram-negative periodontal pathogen) and its proteolytic enzymes called gingipains can translocate to the brain (Dominy et al., 2019). Gingipains are cysteine proteases normally mediating the toxicity of *P. gingivalis* for fibroblasts, endothelial (EC) and epithelial cells (Sheets et al., 2005; Stathopoulou et al., 2009; Kinane et al., 2012). However, Adams et al. have reported that they are also present in the blood of PD patients, suggesting that they can have a role in PD pathology (Adams et al., 2019).

Taking into consideration all the possible communication pathways between the microbiota and the brain that we have described so far, it is quite apparent that the route based on the passage of soluble microbial secretome from the microbiota to brain cells is actually the most reproducible pathway to model the MGBA *in vitro*. Nowadays, organ-on-a-chip technology represents a promising strategy to model this multi-organ communication and researchers have mainly focused on mimicking the MGBA by exploiting several *in vitro* models connected to each other to allow the diffusion of soluble factors and/or metabolites. Because of their complex structure and functional mechanisms, the BBB and the brain are very challenging to model for today's technological tools. For instance, the need for three-dimensional (3D) perfused cell cultures and the difficulties of managing co-cultures of different cell populations are just examples of this complexity when modeling these two human compartments. Thus, if the main purpose is to study the effects of the secretome produced by the gut microbiota on the BBB and the brain, we have to develop more advanced and reliable systems to overcome the technical weaknesses that hinder the modeling of multi-organ communication.

Organs-on-a-chip offer several advantages (Bhatia and Ingber, 2014; Ingber, 2016). They provide spatially-oriented cell-cell interactions and exposure to physical factors, such as fluid flow, shear stress and strain. By perfusing the medium inside the culture chambers, they guarantee a physiological exchange of nutrients and metabolites and shear stress values suitable to stimulate cell growth, proliferation, and differentiation. Furthermore, the miniaturization reduces the amount of reagents needed, and the possibility of integration with electronic devices (e.g., electrodes, sensors) allows to measure biological and physical parameters (e.g., cell viability, trans-epithelial electrical resistance, oxygen pressure, and pH) while in culture.

The literature describes several organs-on-a-chip modeling the human brain and the BBB. In the following paragraphs, we will present the forefront devices and their peculiar features, highlighting their great potential, but also their current limitations.

## Modeling the Brain: Focus on Organ-on-a-Chip-Based *in vitro* Models

The brain is the central organ of the nervous system. It is protected by the skull and other anatomical structures, such as the BBB and the meninges, a layer of membranes covering the CNS. However, pathogens can reach the brain when inflammation alters these protective barriers.

The brain controls all body functions and integrates information from the environment. It comprises several structures mainly made up of two cell populations: nerve cells (neurons) and glial cells.

Neurons are composed of a cell body, dendrites (picking up the messages from other nerves), and an axon (transmitting impulses from the cell body to the periphery). They communicate by several connections (synapses) that allow the passage of nerve impulses. The neuronal circuitry is composed of afferent and efferent pathways that send information by chemical or electrical signals. In chemical synapses, the signals propagate by the release of neurotransmitters, while electrical signals are due to action potentials caused by different ions crossing the neuronal membrane. They are important for the transmission of information that exploits currents flowing along the membrane of the axon to the synapses (Kandel et al., 2012).

Glial cells provide protection, nourishment and structural support to neurons. They comprise astrocytes, microglia, oligodendrocytes, and ependymal cells. For instance, astrocytes play a key role in promoting neuronal survival and maintaining brain homeostasis and the structure of the BBB. Microglia have immune system-like activity. They protect the brain from infections and clean up cell residues (e.g., debris). Oligodendrocytes are essential for neuronal myelination and they contribute to the regulation of the concentration of extracellular ions. However, they are rarely included in *in vitro* models (Jäckel et al., 2017). Ependymal cells are important for the production of cerebrospinal fluid and other substances, but they are included in *in vitro* models only when they are the focus of the model itself (Jäckel et al., 2017). For instance, frequently models focusing on the neurovascular unit (NVU) include ependymal cells.

Researchers have studied brain structure and functions extensively, but including the different cell populations and considering its surface dimensions when modeling the entire brain is an enormous challenge. They have started with single cell cultures, neglecting the possible interactions between neurons and glial cells. More recently, they have moved to the study of cell-cell interactions and improved the mimicking of natural tissue in both physiological and pathological conditions. For instance, co-cultures have supported the study of physical communication between cells (Skaper and Facci, 2012, 2018), Transwell® permeable supports have been used to investigate direct cellular communication in the absence of physical contact and conditioned medium has allowed to assess cell interactions in the absence of physical contact by examining the biochemical factors released (Yoshida et al., 1995; Lin et al., 2016).

3D cultures have contributed to address innovative treatments and enhance clinical translation (Shamir and Ewald, 2014; Hopkins et al., 2015; Hasan and Berdichevsky, 2016). For instance, Tang-Schomer et al. (2014) have modeled brain cortical architecture and reproduced the compartmentalization of gray and white matter by coupling adhesive-free, concentric silk protein-based porous layers and a collagen gel to support 3D axon connections. Lozano et al. (2015) have also proposed a layer-based approach to fabricate 3D brain-like structures by bioprinting gellan gum (conjugated to RGD peptide) with primary cortical neurons. In the context of 3D neural tissue models (Zhuang et al., 2018), bioprinting has demonstrated its potential for the fabrication of constructs embedding glial cells, neurons and stem cells (e.g., Lee et al., 2009; Hsieh et al., 2015; Lozano et al., 2015; Dai et al., 2016; Gu et al., 2016), although the selection of printable materials, cytocompatible with neural cells and able to mimic the mechanical properties of neural tissue while avoiding structural collapse is often difficult.

Thanks to their communicating chambers and dynamic perfusion, organs-on-a-chip are essential tools to support the survival and development of brain tissue, assess cell migration, the direction of axonal extension, transport, and signal transmission.

For instance, Park et al. have proposed a microfluidic chip for the interstitial perfusion of neurospheroids with flow conditions comparable to those in the brain (about 0.1–0.3 μL/min) and applied as an *in vitro* model of AD by testing the toxicity of Aβ. Spheroids (but also organoids grown from stem cells) are powerful 3D biological platforms to investigate neuronal development, drug transport, and the pathogenesis of neuronal diseases. They rely on the capability of small cell aggregates to create *in vitro*, without any existing pattern or foreign material, polarized floating structures similar to *in vivo* tissues. They recapitulate more complex cell-cell interactions and have a higher level of functionality (Fennema et al., 2013; Lancaster et al., 2013; McCracken et al., 2014; Dingle et al., 2015; Paşca et al., 2015), but they lack vascularization and cells hardly reach maturation in culture. Park et al. have fabricated their device by bonding cubic poly(dimethylsiloxane) (PDMS) chambers (1 × 1 × 1 cm) by soft lithography. The top part was dedicated to the flow of culture medium, while the bottom one contained an array structure of fifty cylindrical wells (diameter: 600 μm; height: 400 μm) hosting the neurospheroids. An osmotic micro-pumping system exposed the neurospheroids to a constant flow (about 0.15 μL/min). Their results have indicated that the interstitial flow influences the size distribution of neurospheroids, accelerates the differentiation of neural progenitor cells into neurons, and enhances the proliferation of neural progenitor cells and the toxicity of Aβ with respect to static conditions (Park et al., 2015).

Similarly, Wang et al. have developed a microfluidic chip to obtain human-induced pluripotent stem cell (hiPS)-derived brain organoids to study neurodevelopmental disorders at early stages of gestation. They have used conventional soft lithography to fabricate a PDMS-based device with five independent channels. A central perfusion channel (1 mm wide, 20 mm long) separated two culture channels for the formation and culture of brain organoids (2.5 mm wide, 14 mm long). Embryonic bodies were immobilized in Matrigel and infused into the culture chambers, while medium in static conditions filled the two remaining external channels. Their results have confirmed that organs-on-a-chip provide a controllable microenvironment for an efficient development, maturation, and extended growth of brain organoids (Wang et al., 2018).

Kilic et al. have used organs-on-a-chip to extend culture time, provide a controllable and reliable microenvironment for the differentiation of human pluripotent cells (hPSC) into neuronal and astroglial cells, and study cell migration in response to gradients of chemotactic cues. Their device showed three PDMS layers attached to a glass substrate for mechanical support and high-resolution imaging. The first PDMS sheet modeled the lumens of blood vessels (vascular compartment) and exhibited support pillars to avoid membrane collapse, the second substrate had a porous PDMS membrane (pore size: 5 μm) as a scaffold for the BBB and the remaining PDMS substrate modeled the neural tissue (neuronal compartment). The top and bottom compartments had four perfusion channels (two inlets and two outlets for both chambers; 5 mm wide, 20 mm long, and 300 μm high) (Kilic et al., 2016).

Coupling the BBB to the brain in a single organ-on-a-chip represents a further improvement toward the development of a complete miniaturized model for drug screening and toxicity. For instance, Koo et al. have exploited a device named OrganoPlate® (MIMETAS, The Netherlands). They have filled the brain chamber with a collagen hydrogel embedding N2a neuroblastoma cells, C8D1A immortalized astrocytes and BV-2 immortalized microglia. After gelation, they have defined the BBB compartment by plating bEnd.3 EC facing the hydrogel. However, in this model a rocker shaker (instead of a micro-pumping system) ensured fluid flow and shear stresses and fluid flow, and the Authors had to refresh the medium every 2 days (Koo et al., 2018).

Excitability is a key feature of brain cells, therefore the integration of organ-on-a-chip technology with electrodes is fundamental to both stimulate and read-out the burst-firing power and its frequency rate within the constructs. For instance, Soscia et al. have described removable inserts to deposit neurons from different brain areas (e.g., primary rodent hippocampal and cortical neurons) onto defined regions of a commercial microelectrode array (MEA), without the need of physical or chemical barriers. Their system is a miniaturized device lacking perfusion, but the Authors have proposed an effective method to separate distinct neuronal populations on microfabricated devices, and it offers a unique approach to develop a complex cell environment for anatomically-relevant brain-on-a-chip devices (Soscia et al., 2017).

## Modeling the Blood-Brain Barrier: Focus on Organ-on-a-Chip-Based *in vitro* Models

The properties of the BBB are mainly due to the presence of EC and their interactions with mural, immune, glial and neural cells that in turn interact with the NVU (Abbott et al., 2012; Muoio et al., 2014; McConnell et al., 2017). It is made up of neurons, astrocytes, oligodendrocytes, microglia, smooth muscle cells, brain EC and pericytes embedded in the brain extra-cellular matrix (ECM) and it supports the neuronal circuitry by controlling the permeability of the BBB, the cerebral blood flow and maintaining the chemical composition of the brain interstitial fluid (Zlokovic, 2011).

Transport across the BBB is crucial to maintain brain homeostasis and occurs by specific and selective mechanisms. The phospholipid bilayer of the plasma membrane permits the passage of small gaseous molecules, lipophilic agents and small polar but uncharged molecules (such as ethanol and H_2_O), while specific transporters (e.g., solute carriers and ABC transporters like P-glycoprotein) allow the transfer of hydrophilic molecules (such as glucose). Larger hydrophilic molecules translocate by endocytosis, while the TJ between the EC lining the microvessels of the CNS regulate the paracellular pathway for the movement of ions and molecules in-between cells. In particular, the transmembrane proteins of TJ (e.g., occludins, claudins, and junctional adhesion molecules) are anchored inside the EC by another protein complex, including zonula occludens-1 (ZO-1, also known as tight junction protein-1) and associated proteins (Stamatovic et al., 2008).

In the field of toxicology and drug development against neurological disorders, a number of complex models have been proposed to recapitulate the basic features of the BBB *in vitro*. They have provided insights into the passage of molecules across the BBB and its alterations during disease, cell-cell interactions, and the mechanisms leading to neurodegeneration. Key steps in their validation are transport studies with model molecules and the assessment of the integrity of the barrier by TJ immunostaining and the measurement of the TEER. For mammalian BBBs, the physiological values for the TEER ranges from 1,500 to 2,000 Ω·cm^2^ (Crone and Olesen, 1982; Butt et al., 1990).

Because of its implications in the development of drugs for CNS applications, another fundamental property of the BBB is its permeability. It can be predicted with several computational tools, and measured with different experimental methods. For instance, it is measured with hydrophilic tracers such as sodium fluorescein (376 Da), Lucifer yellow (444 Da), FITC-labeled sucrose, mannitol or dextrans, inulin and bovine serum albumin (Banerjee et al., 2016). In rat models, the permeability of the BBB to sucrose can be as low as 0.03·10^−6^ cm·s^−1^ (Bickel, 2005). For a given small hydrophilic molecule, a correlation exists between the TEER and permeability, but it depends on the size of the molecule and on experimental factors (e.g., shaking, single point estimation vs. steady-state calculations) (Helms et al., 2016).

*In vitro* models of the BBB exploit immortalized or primary brain EC from different animal species, but currently none exhibits a TEER in the physiological range. Primary cultures of human brain EC would be the ideal choice for drug development and preclinical studies, but their limited availability strongly hinders their application. Models based on primary bovine or porcine EC are used to study the transport of small molecules, but the TEER is lower than the physiological ones (Jiang et al., 2019). They can be used for screening studies because their animal sources allow the harvesting of a great number of cells, but the pattern of protein expression differs from that of the corresponding human proteins and in some cases, the experimental barrier shows different affinity and transport rates with respect to the human BBB. Mouse or rat brain EC are easier to obtain, but the low yield makes their routinely use impractical. For these reasons, the great majority of models is based on mouse EC lines (e.g., bEnd.3, bEnd.5, immortalized mouse cerebral capillary EC, cEND). Human immortalized EC (e.g., CMEC/D3 cells) are also commercially available and they are suitable to study transporters, receptors, signaling pathways and metabolism (Helms et al., 2016). As an alternative, other models exploit self-renewable cells (such as hPSC) that can differentiate into mature somatic cells. Some of these models has provided a good approximation of the physiological BBB, but actually, they are still far to mimic the changes in permeability and drug permeation found in several pathological conditions. Table 2 lists recent two-dimensional (2D) co-cultures based on immortalized or primary cells and it reports their distinctive hallmarks (junction proteins, TEER, permeability). Similarly, Table 3 shows examples of models based on self-renewable cells.

**Table 2:** *In vitro* models of the BBB, 2D condition.

**Cell populations**	**2D models**	**Disease**	**Junction proteins**	**~TEER (Ω·cm^**2**^)** **P**_**e**_**10**^**−6**^ **(cm·s**^**−1**^)	**References**
Primary mouse BCEC + astrocytes	co-culture	–	occludins, claudins 3 and 5	TEER 800 P_e_ 4.5 (with sucrose)	Coisne et al., 2005
Primary rat BCEC + astrocytes	co-culture	AD, encephalitis, MS	occludins, claudins 5, ZO-I	TEER 300–600 P_e_ 1.4 (with sucrose) P_e_ 4.3 (with Lucifer yellow)	Nakagawa et al., 2007, 2009; Perrière et al., 2007; Abbott et al., 2012
Primary rat BCEC + astrocytes + pericytes	co-culture	AD	occludins, claudins 5, ZO-I	TEER 350–723 P_e_ 2–4 (with sodium fluorescein)	Nakagawa et al., 2007, 2009; Veszelka et al., 2013; Walter et al., 2015
Primary rat BCEC + astrocytes + neurons	co-culture	AD, epilepsy	occludins, ZO-I	TEER 268	Xue et al., 2013
Primary bovine BCEC + rat astrocytes	co-culture	–	Occludins claudins 1 and 5	TEER 600–800 P_e_ 0.5 (with mannitol)	Gaillard et al., 2000, 2001; Helms et al., 2010; Helms and Brodin, 2014
Primary bovine BCEC (clonal selection) + rat astrocytes	co-culture	–	occludins, claudins 1 and 5, ZO-I	TEER 800 P_e_ 6–12.5 (with sucrose)	Dehouck et al., 1990; Cecchelli et al., 1999; Vandenhaute et al., 2011
Primary porcine BCEC + rat astrocytes or astrocyte cell line	co-culture	AD, HD	occludins, claudins 5, ZO-I	TEER 800–1,800 P_e_ 0.6 (with Lucifer yellow)	Cohen-Kashi Malina et al., 2009; Cantrill et al., 2012; Patabendige et al., 2013

They are based on primary or immortalized cells.

*AD, Alzheimer's disease; BCEC, brain capillary endothelial cells; HD, Huntington's disease; MS, multiple sclerosis; P_e_, permeability; TEER, trans-endothelial electrical resistance; ZO-1, zonula occludens-1*.

**Table 3:** *In vitro* models of the BBB, 2D condition.

**Cell populations**	**2D models**	**Disease**	**Junction proteins**	**~TEER (Ω·cm^**2**^)** **P**_**e**_**10**^**−6**^ **(cm·s**^**−1**^**)**	**References**
hPSC + rat astrocytes	co-culture	AD, MS and brain tumors	occludins, claudins 5, ZO-I	TEER 700 P_e_ 0.6 (with sucrose)	Lippmann et al., 2012
hPSC + pericyte-primed human NPC	co-culture		occludins, claudins 5, ZO-I	TEER 5350 P_e_ 0.6 (with sucrose)	Lippmann et al., 2014
Cord blood-derived endothelial progenitor cells + pericytes	co-culture	AD, MS	occludins, claudins 5, ZO-I	TEER 160 P_e_ 10–20 (with Lucifer yellow)	Cecchelli et al., 1999; Boyer-Di Ponio et al., 2014
hIPS-EC + hIPS-NSC + astrocytes + pericytes	co-culture	AD, PD	occludins, claudins 3, 4 and 5 ZO-I	TEER 433–2,489 P_e_ 1.58 (with Lucifer yellow) P_e_ 1.33 (with sodium fluorescein)	Appelt-Menzel et al., 2017

They are based on human-induced pluripotent stem (hIPS) cells or progenitor cells.

*AD, Alzheimer's disease; hIPS-EC, human-induced pluripotent stem cell-derived BBB endothelial cells; hPSC, human pluripotent stem cells; MS, multiple sclerosis; NPC, neural progenitor cells; NSC, neural stem cells; PD, Parkinson's disease; P_e_, permeability; TEER, trans-endothelial resistance; ZO-1, zonula occludens-1*.

Both tables refer to static conditions based on the Transwell® system, where a microporous semipermeable membrane is suspended in the culture wells. It allows the diffusion of molecules from one side to another and it separates the vascular compartment (EC) from the parenchymal one (e.g., astrocytes). According to the complexity of the model, the cell populations are plated on the plastic well and on the top side of the membrane, on the plastic well and on both sides of the membrane or only on both sides of the membrane (Bors and Erdö, 2019).

Dynamic *in vitro* models of the BBB offer several advantages over conventional 2D systems, but they require a higher flow rate than in physiological conditions because of the larger volume of the microtubes with respect to brain capillaries. As for the static models, they comprise two chambers (mimicking the vascular and parenchymal compartments) separated by a microporous semipermeable membrane, acting as a physical barrier.

Griep et al. have modeled the BBB to study barrier dysfunctions in neurodegenerative conditions. Their chip was made up of two PDMS layers divided by a Transwell®-like polycarbonate membrane (pore size: 0.4 μm, thickness: 10 μm) to culture CMEC/D3 EC. To measure the TEER, the Author haves included Pt electrodes inside furrows in the PDMS sheets after assembling the top and bottom parts of the chip (Griep et al., 2013).

Booth and Kim have proposed a more complex microfluidic model of the BBB to study its function and drug delivery. They have developed a multi-layered and fully integrated device, made up of (from top to bottom) a PDMS sheet, a glass electrode layer, a PDMS substrate, a porous polycarbonate membrane for cell culture, another PDMS layer, a glass electrode substrate and finally a last PDMS sheet. The two central PDMS layers hosted the perfusion channels (named as abluminal, the inferior one; and luminal, the superior one). They were perpendicular and their aspect ratios were designed to induce a uniform shear stress distribution on bEnd.3 EC (for the abluminal channel) and minimize the shear stresses on C8D1A astrocytes (for the luminal channel). The electrodes in the glass substrates opposite to the membrane allowed to monitor the TEER. To reduce the noise due to the wiring resistance, the Authors have used two sets of two thin-film AgCl electrodes forming a four point sensing structure (Booth and Kim, 2012). However, the electrodes covered about 75% of the culture surface, limiting the optical accessibility and thus the real-time monitoring of cell constructs.

When designing an organ-on-a-chip model of the BBB with both a vascular and parenchymal compartment, assembly is a critical step. In fact, the membrane needs to be seeded on both sides and turned upside down after the first seeding. Brown et al. have reported an interesting solution to tackle this challenge in a complex device, based on the co-culture of four cell populations and equipped with independent inlets and outlets to perfuse the chambers with different media. Their microfluidic chip was composed of three PDMS layers with a 0.2 μm polycarbonate membrane dividing the first PDMS sheet from the others. In the first PDMS layer, they have plated primary human brain-derived microvascular EC on the bottom of the membrane and perfused with their medium. Twelve days later, they have turned upside down the device and plated primary astrocytes and pericytes on the other side of the membrane. To avoid the overturning of the inlet and outlet tanks when changing the orientation, they have equipped the device with a flippable backpack. Two days later, they have added cortical glutamatergic neurons from hiPSs in the third PDMS substrate. A further feature of interest of this model is the fact that the Authors have embedded the neurons in a 3D collagen hydrogel to mimic the ECM (Brown et al., 2015).

Adriani et al. have removed the microporous membrane and started from the importance of a 3D matrix mimicking brain ECM. They have proposed a model of the NVU based on a single layer device composed of four parallel PDMS channels. The first channel hosted only medium, the second channel cortical neurons embedded in a collagen hydrogel, the third channel primary astrocytes embedded in a different collagen hydrogel and the last one EC. Each channel communicated directly with the adjacent one, allowing direct cell-cell contact and signaling (Adriani et al., 2017).

In their NVU platform for drug screening applications, Bang et al. have designed separated microchannels to supply different culture media to the cells. Their vascular channel connected to the inner lumen of the vascular network (hosting human fibroblasts and human umbilical vein EC), while the neural channel perfused rat cortical neurons adherent to the vascular network. The Authors have observed a good agreement between the permeability coefficients obtained by 20 and 70 kDa FITC-dextran in their NVU and *in vivo*, suggesting that the use of umbilical cord ECs instead of brain-derived ECs is not a limitation (Bang et al., 2017).

Similarly to Adriani et al., Campisi et al. have highlighted the importance of a 3D matrix mimicking the ECM, but they have also focused on culturing human brain cells to remove cross-species incompatibility and achieve physiologically relevant results. They have filled the ports of their chip with a fibrin hydrogel embedding the co-culture of hiPS-EC, pericytes and astrocytes from human brains and pipetted hiPSC-EC into the channels. The results have suggested that their model shows physiologically relevant structures and is suitable for several applications, such as the study of the neurovascular function, transport across the BBB and metastatic cancer extravasation to the brain (Campisi et al., 2018).

## General Considerations and Challenges for the MGBA *in vitro* Modeling

Information about the MGBA has mainly come from *in vivo* studies on GF and SPF mice. Animal models are suitable to reproduce all the communication pathways involved, but they may differ from humans. Moreover, they exhibit high variability (e.g., in SPF mice the excluded mouse pathogens depend on breeding conditions, Hirayama et al., 1990) and the isolation of a single parameter leading to alterations in the gut microbiota or brain homeostasis is difficult. They also pose ethical and economic issues (Dobson et al., 2019).

In accordance with the 3Rs principle (Replacement, Reduction and Refinement) (Cronin, 2017), *in vitro* models represent a strategy to overcome these limitations and recapitulate fundamental disease mechanisms. Since they are based on a more controllable and reproducible experimental setup, they offer the possibility to isolate and test different parameters. However, their simplicity with respect to the complexity of the real *in vivo* situation limits their effective applicability to the study of the MGBA (Cryan et al., 2019).

2D cultures on plastic surfaces are valuable tools for cell-based studies because of their cost-effectiveness, ease of handling and robustness across different cell types. They allow the diffusion of secreted soluble factors in the medium, but they force cell attachment in a planar direction and prevent cell-cell and cell-ECM interactions, thus affecting proliferation rate and differentiation. In their physiological microenvironment, cells are surrounded by their own 3D matrix; moreover, they are exposed to endocrine signals from distant tissues, paracrine signals from nearby cells and physical stimuli (e.g., shear stress and oxygen tension). In 2D cultures, we lack most of this biological, mechanical and topographical complexity (Paşca, 2018). Assembloids (Paşca, 2018) rely on the controlled assembly of 3D cultures to recapitulate more complex cell-cell interactions (e.g., by mixing cells of different lineages or adding cells and biomaterials with organizer-like capabilities), leading to models with improved tissue architecture and reproducibility. To this purpose, hydrogels are promising tools because of their tunable properties, and bioprinting offers a noteworthy contribution to fabricate 3D living constructs with superior spatial resolution and an automatic and exact cell arrangement (Moroni et al., 2018; Zhuang et al., 2018). However, assembloids need improvements to mimic the ECM (e.g., for brain cultures), support cell viability in larger constructs and control dynamic features (e.g., pH and oxygen levels).

Organs-on-a-chip allow cell culturing in single chambers, promoting their communication by microfluidic channels and controlling the spatial and temporal distribution of their microenvironment. They offer the possibility to establish more complex, physiologically-relevant and reliable conditions by creating gradients, setting the medium flow to ensure the exchange of nutrients and metabolites and stimulate cell growth, proliferation and differentiation, applying mechanical forces to mimic the physical microenvironment of living organs (e.g., peristalsis-like deformations in the gut), and monitoring the operating parameters (e.g., oxygen, glucose concentration and pH) (Bhatia and Ingber, 2014; Ingber, 2016). In particular, the miniaturization reduces the reagent volumes, while the integration of electronic sensors allows measuring biological and physical parameters (e.g., trans-endothelial/trans-epithelial electrical resistance, usually referred to as TEER, a key parameter to evaluate the integrity of physiological barriers, such as the BBB and the gut epithelium). Since they are miniaturized models of the major functional units of whole organs, the use of organs-on-a-chip is currently a very promising strategy to model the MGBA *in vitro*.

By the combination of physiologically-relevant conditions and cutting-edge technological devices, organs-on-a-chip (and their connection into multi-organ platforms) hold potential for supporting research aimed at the comprehension of the molecular mechanisms underlying disorders involving different organs, exploring frontier hypotheses with a multidisciplinary approach and leading to the development of new therapeutic strategies. For instance, Ingberg's research group has provided crucial results for the study of host-microbiome interactions and the development of microbiome-related therapeutics, probiotics and nutraceuticals. In particular, by a microfluidic device ensuring oxygen gradients, Jalili-Firoozinezhad et al. (2019) have co-cultured aerobic and anaerobic human gut microbiota in contact with human intestinal epithelium and its mucus layer on top.

However, the path to model the entire MGBA with a single multi-organ platform is still long. However, from the previous discussion, it is apparent that interdisciplinary strategies combining bioengineering, biochemistry and medicine and leading to innovative *in vitro* tools are required to overcome technical and biological limitations, and develop reliable platforms to study the whole MGBA.

To this respect, a contribution may come from bioprinting and its applications. This is a technological approach for the simultaneous incorporation of materials, cells and biologically active factors (e.g., for haptotactic gradients; Ilkhanizadeh et al., 2007) with high spatial control and biomimicry. It is a cutting-edge technology with remarkable potential in the field of biomaterials and 3D tissue models. It is highly versatile in terms of shape, porosity and control of material interconnectivity, it provides a more automated and defined approach to biomaterial-cell interfaces, thus fabricating structures with custom-made architectures and create layered and viable 3D cell constructs. However, it requires the precise control of the rheological properties of the bioinks (Paxton et al., 2017). Because of their ability to retain water, hydrogels are excellent microenvironments for cell culture. For this reason, current bioinks for brain cells are hydrogels, both from natural and synthetic polymers. Natural polymers are usually preferred because of their intrinsic biocompatibility, but they are more difficult to print than synthetic ones or after printing, their mechanical properties are physiologically irrelevant. Further challenges concern the fact that cell density may influence the final properties of the constructs and stress occurring during printing may affect cell survival and behavior (Moroni et al., 2018; Zhuang et al., 2018).

Despite these limitations, the integration of biomaterials and bioprinting in organs-on-a-chip modeling the brain and the BBB (and, more in general, in *in vitro* tissue models and disease models) would add complexity to recreate the microenvironment by mimicking the composition and anatomical properties of the ECM and providing key mechanical, biochemical and topographical cues as gradients or localized hotspots (Wolf et al., 2019). The relevance of these benefits has strongly emerged in research fields where the development of vascularized structures is of pivotal importance, like cancer research. For instance, a high degree of vascularization is a pathological hallmark of malignant gliomas and cancer cells migrate through microvessels. In this context, the development of more complex and representative tumor-vascular niches would help to assess cell-cell and cell-ECM interactions, model clinically-relevant phenomena (e.g., patient-specific resistance to chemoradiotherapy; Yi et al., 2019) and suggest innovative therapeutic strategies.

The coupling of bioprinting to microfluidics might also drive the development of automated devices for drug screening and toxicity testing. In this context, bioprinting would allow for a rapid fabrication of hydrogel-based cell-embedding constructs with highly controllable and reproducible thickness. This would lead to consistent diffusion across the hydrogel matrix, with more reproducible results across different experiments (Bhise et al., 2016).

Coming back to the issue of modeling the whole MGBA, we would like to bring to attention that recently the European Research Council has funded a project named MINERVA (ID 724734). It aims at applying a bioengineering approach to the MGBA, by developing technological platforms to elucidate the effect of microbiota secretome on brain functions, in a context of AD-related neurodegeneration. The engineered platform will be suitable not only to study the MGBA, but also to examine other pathologies involving several organs.

**Figure 1:**
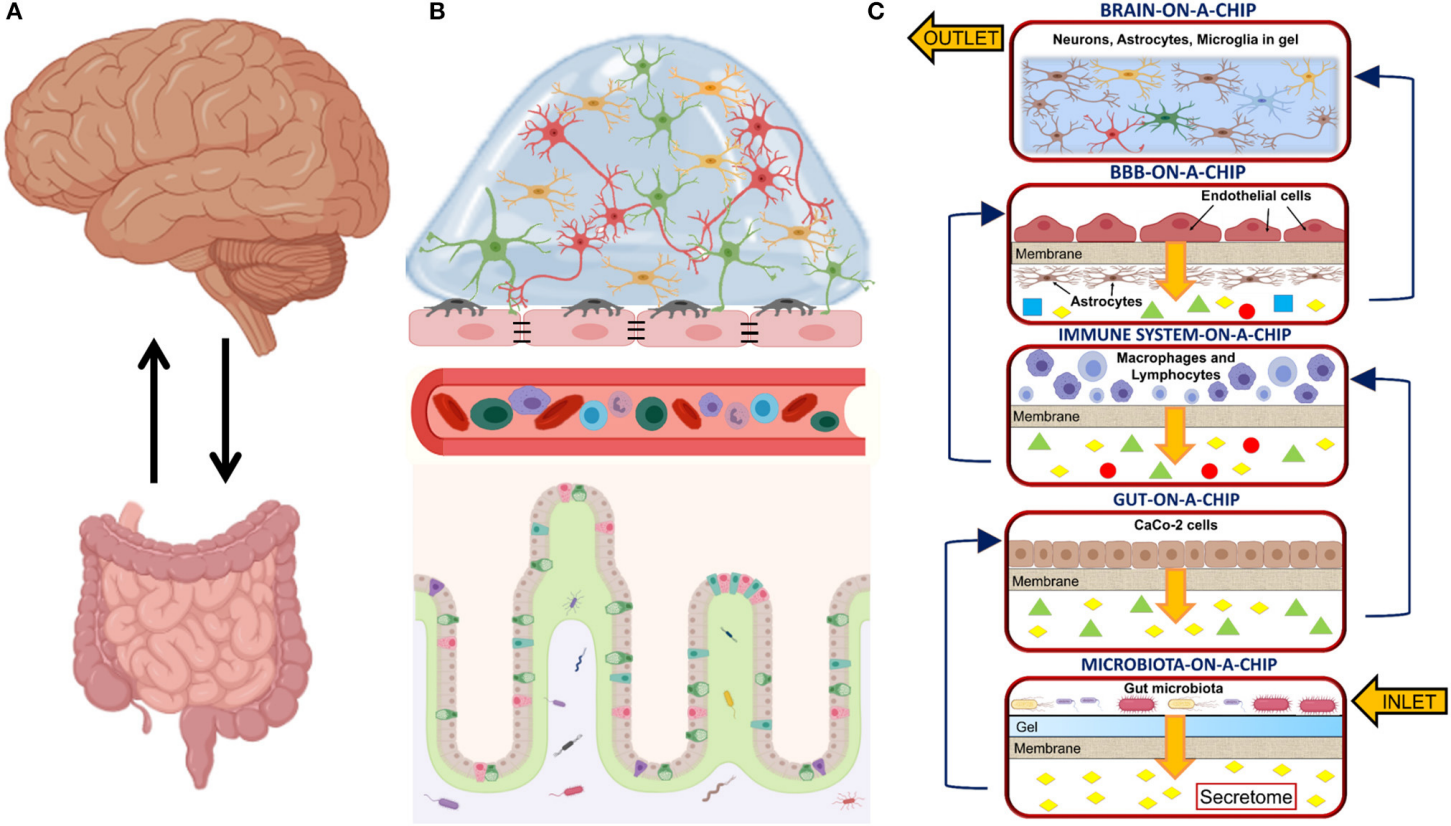
**(A)** Sketch showing the main organs involved in the microbiota-gut-brain axis and their bidirectional communication; **(B)** Sketch detailing the biological structures involved in the microbiota-gut-brain axis. From bottom to top: the microbiota resides in the intestinal lumen and in the loose layer of the intestinal mucus. It interplays with the epithelial cells in the gut epithelium, the cells of the immune system circulating into the bloodstream, the blood-brain barrier (BBB) composed of endothelial cells (EC), pericytes and astrocytic end-feet processes and brain cells (e.g., neurons, microglia, astrocytes, and oligodendrocytes); **(C)** Sketch of the MINERVA organ-on-a-chip platform. From bottom to top: the microbiota-on-a-chip device hosts a microporous membrane supporting a hydrogel-based matrix mimicking gut mucus and inoculated with gut microbiota; the gut-on-a-chip device hosts a microporous membrane seeded with gut epithelial cells (e.g., CaCo-2 cells); the immune system-on-a-chip device hosts macrophages and lymphocytes; the BBB-on-a-chip device hosts two specular monolayers of EC and astrocytes; the brain-on-a-chip device hosts a 3D hydrogel matrix mimicking brain extracellular matrix (ECM) and embedding neurons, microglia and astrocytes. In the MINERVA platform, we have considered two configurations for the brain-on-a-chip device: the first is composed of three chambers hosting the co-culture of neurons, microglia and astrocytes, while the second has three chambers hosting neurons, microglia and astrocytes as a single culture. Both the microbiota and the immune system compartments have filters at the inlet and outlet to prevent cell migrating out from the culture chambers. With the exception of the brain device, each compartment has a microporous membrane to support cell adhesion. It also allows the passage of secretome to the lower part of the culture chamber without mixing of the different culture media. Created with BioRender.com.

The main challenge of the MINERVA project lies in the great complexity arising from the involvement of many biochemical pathways, multi-organ functions and crosstalks. Five optically accessible, microfluidic organs-on-a-chip will compose the final platform. They will model the microbiota, the gut, the immune system, the BBB, and the brain compartment. Each compartment will permit both sampling and cell perfusion. A microporous membrane will divide each chamber into two parts, separating the cells from culture medium flowing to the next device. Therefore, before reaching brain cells, culture medium will be enriched with secretome from the gut microbiota, gut epithelial cells, immune cells and BBB cells (Figure 1). A hydrogel matrix simulating the intestinal mucus and hosting the gut microbiota (e.g., from fecal samples from healthy donors or AD patients) will populate the first device and an adjacent flow of medium will collect the secretome. In the following device, gut epithelial cells will be plated on the microporous membrane to model the gut barrier. The next device will host both lymphocytes and macrophages to model the immune system and a microporous membrane will prevent their exit from the device. To model the BBB, EC and astrocytes will be plated on both sides of a microporous membrane; while the final device will model the brain. A 3D hydrogel modeling brain ECM will embed neurons, astrocytes and microglia (as single cell cultures or as a co-culture. For the design and development of the basic organ-on-a-chip device, MINERVA has started from a miniaturized, optically accessible bioreactor developed for the interstitial perfusion of 3D cell constructs (Izzo et al., 2019) and validated for advanced *in vitro* cell modeling (Tunesi et al., 2016; Marturano-Kruik et al., 2018). MINERVA may be the first example of a multi-organ platform suitable to face *in vitro* with a very complex biochemical pathway as the MGBA, paving the way to significant advancements in the field, for example the discovery of new, not invasive therapies for neurodegenerative disorders based on microbiota management by food ingredients or probiotics.

## Author Contributions

IR, LI, and MT wrote, reviewed, and edited the original draft. MC reviewed the original draft. DA reviewed and edited the original draft. CG reviewed and edited the manuscript.

### Conflict of Interest

The authors declare that the research was conducted in the absence of any commercial or financial relationships that could be construed as a potential conflict of interest.

## Abbreviations

2D: two-dimensional
3D: three-dimensional
5-HT: 5-hydroxytryptamine (serotonin)
α-syn: α-synuclein
Aβ: β-amyloid
AD: Alzheimer's disease
ALS: amyotrophic lateral sclerosis
ANS: autonomic nervous system
ASD: autism spectrum disorders
BBB: blood-brain barrier
BCEC: brain capillary endothelial cells
BMAA: β-methylamino-L-alanine
CNS: central nervous system
cEND: cerebral capillary endothelial cells
EC: endothelial cells
ECM: extracellular matrix
ENS: enteric nervous system
GABA: γ-aminobutyric acid
GI: gastrointestinal
GF: germ-free
HD: Huntington's disease
HPA: hypothalamic-pituitary-adrenal axis
hPSC: human pluripotent stem cells
hIPS cells: human-induced pluripotent stem cells
LPS: lipopolysaccharide
MEA: multielectrode array
MGBA: microbiota-gut-brain axis
MOAB: miniaturized optically accessible bioreactor
MS: multiple sclerosis
NPC: neural progenitor cells
NSC: neural stem cells
NVU: neurovascular unit
PD: Parkinson's disease
PDMS: polydimethylsiloxane
P_e_: permeability
SCFA: short-chain fatty acids
SPF: specific pathogen-free
TEER: trans-endothelial electrical resistance or trans-epithelial electrical resistance
Thy-1: thymocyte differentiation antigen 1
TJ: tight junctions
ZO-1: zonula occludens-1.
